# Detection of Polioviruses Type 2 among Migrant Children Arriving to the Russian Federation from a Country with a Registered Poliomyelitis Outbreak

**DOI:** 10.3390/vaccines12070718

**Published:** 2024-06-28

**Authors:** Olga E. Ivanova, Tatiana P. Eremeeva, Olga Y. Baykova, Alexandr Y. Krasota, Elizaveta V. Yakovchuk, Elena Y. Shustova, Lyudmila P. Malyshkina, Aida N.-I. Mustafina, Yulia M. Mikhailova, Alina V. Chirova, Evgeniya A. Cherepanova, Nadezhda S. Morozova, Anna S. Gladkikh, Anna S. Dolgova, Vladimir G. Dedkov, Areg A. Totolian, Liubov I. Kozlovskaya

**Affiliations:** 1 Federal State Autonomous Scientific Institution “Chumakov Federal Center for Research and Development of Immune-and-Biological Products of the Russian Academy of Sciences” (Institute of Poliomyelitis) (FSASI “Chumakov FSC R&D IBP RAS”), 108819 Moscow, Russia; poliom_ldms@mail.ru (T.P.E.); baykovaaa@mail.ru (O.Y.B.); yakovchuklisa@gmail.com (E.V.Y.); riw.sun@list.ru (E.Y.S.);; 2 Department of Organization and Technology of Production of Immunobiological Preparations, Institute for Translational Medicine and Biotechnology, First Moscow State Medical University (Sechenov University), 119048 Moscow, Russia; 3 Federal Budgetary Health Institution “Federal Center of Hygiene and Epidemiology” of the Federal Office for Inspectorate in the Field of Customers and Human Well-Being Protection” (FBHI FCH&E), 117105 Moscow, Russia; mikhailovaym@fcgie.ru (Y.M.M.); chirovaav@fcgie.ru (A.V.C.); cherepanovaea@fcgie.ru (E.A.C.); morozovans@fcgie.ru (N.S.M.); 4 Saint-Petersburg Pasteur Institute, Federal Service on Consumers’ Rights Protection and Human Well-Being Surveillance, 197101 Saint-Petersburg, Russia; gladkikh@pasteurorg.ru (A.S.G.); annadolgova@inbox.ru (A.S.D.); vgdedkov@yandex.ru (V.G.D.); totolian@spbraaci.ru (A.A.T.); 5 Martsinovsky Institute of Medical Parasitology, Tropical and Vector Borne Diseases, First Moscow State Medical University (Sechenov University), 119048 Moscow, Russia

**Keywords:** poliomyelitis, poliovirus vaccine, novel oral poliovirus vaccine type 2, nOPV2, vaccine derived poliovirus, VDPV2, migrants

## Abstract

The widespread use of the oral poliovaccine from Sabin strains (tOPV) radically reduced poliomyelitis incidence worldwide. However, OPV became a source of neurovirulent vaccine-derived polioviruses (VDPVs). Currently, circulating type 2 VDPVs (cVDPV2) are the leading cause of poliomyelitis. The novel OPV type 2 vaccine (nOPV2), based on genetically modified Sabin strain with increased genetic stability and reduced risk of cVDPV formation, has been used to combat cVDPV2 outbreaks, including one in Tajikistan in 2021. In order to identify the importation of cVDPV2 and nOPV2-derivates, stool samples from 12,127 healthy migrant children under 5 years of age arriving from Tajikistan were examined in Russia (March 2021–April 2022). Viruses were isolated in cell culture and identified via intratype differentiation RT-PCR, VP1 and whole-genome sequencing. cVDPV2 isolates closely related with the Tajikistan one were isolated from two children, and nOPV2-derived viruses were detected in specimens from 106 children from 37 regions of Russia. The duration of nOPV2 excretion ranged from 24 to 124 days post-vaccination. nOPV2 isolates contained 27 mutations per genome (0.36%) on average, with no critical genetic changes, which confirms the genetic stability of nOPV2 during field use. The possibility of epidemiologically significant poliovirus introduction into polio-free countries has been confirmed. The screening of special populations, including migrants, is required to maintain epidemiological well-being.

## 1. Introduction

The properties of the oral trivalent poliovirus vaccine from Sabin strains (tOPV), most importantly, the ability to induce mucosal immunity to help interrupt poliovirus (PV) circulation, has made it the optimal vaccine for the realization of the Global Polio Eradication Initiative (GPEI) of the World Health Organization (WHO) [1]. The known disadvantages of tOPV are the ability to cause extremely rare cases of vaccine-associated paralytic poliomyelitis (VAPP) and the ability of Sabin strains to transform into neurovirulent variants (vaccine-derived polioviruses, VDPV) under certain circumstances [2]. However, these disadvantages cannot outweigh the role of the vaccine in the fight against wild PVs. The use of tOPV has achieved very impressive results: the circulation of indigenous wild PVs was stopped in 5 out of 6 WHO regions, wild PV types 2 and 3 have been eradicated worldwide, only two countries (Afghanistan and Pakistan) remain endemic for polio and only wild PV type 1 is circulating [2]. Nevertheless, as the number of polio cases associated with wild PVs declines, the disadvantages of tOPV have become a serious problem. The occurrence of post-vaccination complications in the form of VAPP is unacceptable in the absence of polio cases caused by wild PVs, and it discredits the vaccine prevention system as a whole. Moreover, the most significant obstacle is VDPVs, primarily circulating VDPVs type 2 (cVDPV2), which are responsible for the most polio cases and outbreaks in the world currently [3]. The WHO’s decision to globally stop the use of tOPV in April 2016 (“switch”) and replace it in routine immunization with bivalent OPV (bOPV) types 1 and 3 was based on the fact that wild PV2 had not been detected since 1999 and aimed to stop the circulation of VDPV2 [2]. To maintain population immunity to PV2 after the “switch,” the introduction of at least one dose of trivalent inactivated poliovirus vaccine (IPV) into routine immunization in all countries was proposed [4]. However, in some countries, the introduction of IPV was postponed for several years, which led to a deficiency of population immunity to PV2 [5]. Monovalent OPV type 2 (mOPV2) from the Sabin2 strain was chosen as the main tool for responding to cVDPV2 outbreaks [5]. Due to a number of reasons, primarily the unsatisfactory quality of immunization campaigns in response to outbreaks, the number of polio cases caused by cVDPV2 increased dramatically: in the period of 2016–2020, 109 VDPV2 outbreaks were reported in 34 countries in four WHO regions [6]. The vast majority of viruses were mOPV2 derivatives [5], i.e., response measures with Sabin OPV triggered new VDPV2 genetic lineages [6]. The solution to the problem seemed to be a novel OPV2 from a genetically modified Sabin2 strain (nOPV2) [7], with more stable genetic profile and decreased recombination capacity but restored high immunogenicity [2,8]. In 2020, nOPV2 was approved by the WHO for emergency use [9], and in 2023, the WHO issued a prequalification approval for nOPV2. Almost 1 billion doses have been used to date in 35 countries [10]. This vaccine was used in the Republic of Tajikistan in response to an outbreak associated with imported cVDPV2 from Pakistan in 2020–2021 [11]. The outbreak continued until July 2021, and 36 poliomyelitis cases were registered [12]. The outbreak was attributed to the delay in the IPV introduction, which had led to the formation of a significant group of children lacking immunity to PV2, especially among those who were born after the “switch” [12,13]. Vaccination activities to interrupt the outbreak included 1 round of vaccination with IPV and 3 rounds with nOPV2 in 31 May–6 June 2021; 29 June–3 July 2021; and 30 August–4 September [11,12].

Events of the importation of wild PVs or VDPVs into Russia from other countries, including by healthy people without clinical manifestations, have been repeatedly recorded [14,15,16,17]. A significant number of labor migrants from Tajikistan (up to one million, according to various estimates) work in Russia. In accordance with Russian national documents [18], children under 5 years of age arriving from countries affected by polio are examined for the presence of the poliovirus upon any hospital visit. During the outbreak in Tajikistan in 2021, all children arriving from Tajikistan were subject to virological examination. This decision was justified, among other things, by the fact that VDPV2 was imported from Tajikistan to Ukraine in 2021 [19]. The purpose of the study was not only to identify the possible importation of cVDPV2; nOPV2 isolates that could potentially be imported into Russia by children vaccinated in Tajikistan were of significant interest as well. Here, we present the results of these studies.

## 2. Materials and Methods

### 2.1. Study Design: Collection of Material and Laboratory Research Algorithm

The prospective cohort study was carried out in accordance with the order of the Federal service of surveillance for protection of consumer’s rights and human well-being of the Russian Federation (Rospotrebnadzor). One fecal sample was collected from all children under 5 years of age arriving from Tajikistan after 3 March 2021 until April 2022, when the WHO announced the end of the outbreak in Tajikistan [20]. In 81 regions of Russia, 12,127 children were examined. The investigation of the samples was carried out by the virological laboratories of the Centers for Hygiene and Epidemiology of Rospotrebnadzor in each region of Russia. Virological investigations in RD and L20B cell cultures were performed in accordance with WHO recommendations [21]. The identification of cytopathogenic agents was carried out using RT-PCR AmpliSense Enterovirus-FL kits (Central Research Institute of Epidemiology of Rospotrebnadzor, Moscow, Russia). All L20B-positive or enterovirus isolates or unidentified isolates and in some cases (for example, in the absence of virological laboratory in the region, or if the laboratory could not cope with the volume of incoming stool samples, in some cases—to speed up the study), stool samples were sent to the National Laboratory for Polio (NL) at the “Chumakov FSC R&D IBP RAS” (Institute of Poliomyelitis) for identification. Intratypic differentiation (ITD) was performed with a RT-PCR kit [22] and an additional set of primers for nOPV2 identification (CDC, Atlanta, GA, USA). In the case of the detection of a PV2-positive sample, the child’s specimens were collected every 1–3 weeks until a negative result was obtained, but this proved difficult to organize as migrants moved away or changed addresses. Taking into account repeated sampling, 12,500 stool samples were examined.

A vaccination history was collected from all children: vaccination against polio, the date of the last vaccination and the type of vaccine, including the date of nOPV2 vaccination, and the date of entry to Russia.

### 2.2. VP1 Sequencing

RNA was extracted from cultural isolates using the RNeasy Mini Kit (Qiagen, Hilden, Germany), reverse-transcribed with SuperScript reverse transcriptase (Invitrogen, CA, USA) and amplified by PCR [23]. PCR fragments were gel-purified and extracted with a QiaQuick DNA purification kit (Qiagen, Hilden, Germany). For sequencing, an ABI 3130 Genetic Analyzer was used.

### 2.3. High Throughput Sequencing

RNA was extracted from cultural isolates with a QIAamp Viral RNA Extraction Kit (QIAGEN, Hilden, Germany) in accordance with the manufacturer’s protocol. Total RNA was used for library preparation using NEBNext Ultra II RNA Library Preparation Kit for Illumina. After adapter ligation, libraries were purified with NEBNext Sample Purification Beads. Library quality was assessed using capillary electrophoresis with Qiaxcel Advanced (QIAGEN, Hilden, Germany); the resulting libraries had size of about 300 bp. The concentration was determined using a Qubit-4 fluorimeter. Libraries with unique indexes were pooled in equimolar ratios and sequenced using MiSeq v3 chemistry with 150 bp paired end sequencing. The quality of Illumina readings was assessed using the FastQC program. Raw reads were filtered and trimmed with Trimmomatic (PE mode, ver. 0.39) [24]. Trimmed reads were mapped to the Human poliovirus 2 type nOPV2 (GenBank ID MZ245455) as a reference genome with bowtie2 (v.2.3.5.1) [25] in local alignment mode. All reads were then assigned to read groups by Picard Toolkit (ver. 2.27.4, Broad Institute, https://broadinstitute.github.io/picard/ accessed on 7 August 2023). Variant calls were performed with GATK (ver. 4.2.6.1, Broad Institute) [26]. The SAMsools and bcftools software were used to generate consensus [27].

### 2.4. Classification of nOPV2 Isolates

Sequences of nOPV2 isolates were compared to the sequences of the nOPV2 vaccine strain (GenBank ID MZ245455) and classified on a scale from 1 to 9 based on their risk profile and loss of key attenuating mutations as described [28]. The scale takes into account the presence of mutations compared to the sequence of the nOPV2 vaccine strain, the replacement of the V domain and the cre-element in the 5′UTR, possible recombination with other enteroviruses and the number of mutations in the VP1 protein.

### 2.5. Research Ethics

Parents or guardians of participants were notified of the study’s purposes. Since the study was conducted in accordance with the Polio and Acute Flaccid Paralysis (AFP) Surveillance Program in Russia, specific informed consent was not required.

## 3. Results

A total of 1325 different materials (745 isolates, 580 fecal samples) were examined at NL during the study. Polioviruses were isolated from 109 children: nOPV2 derivatives were detected in samples from 106 children from 37 regions of Russia; two children from 2 different regions excreted VDPV2, and one child excreted a mixture of Sabin-like polioviruses types 1 and 3. The average age of virus excretors was 2.8 ± 1.95 years, and gender was known for 92 children (40 female, 52 male).

### 3.1. VDPV2 Isolation

In the first case, VDPV2 was isolated from a healthy 3-year-old girl who received a single dose of bOPV on 8 September 2018; according to her parents, she did not receive nOPV2 (Table 1). The girl entered the territory of Russia on 29 July 2021, and a fecal sample was collected on 3 September 2021. After the isolation of VDPV2, fecal samples were collected from the child several times (8 times). VDPV2 was isolated from 5 samples (collected from 3 September 2021 to 22 October 2021), and 3 subsequent samples (sampled from 29 October 2021 to 17 November 2021) were negative. Thus, the time of VDPV2 excretion from the moment of entry into the territory of Russia was 85 days, and within 45 days (from the moment of entry to the laboratory result on 7 September 2021), polio remained undetected in excretion.

The second case of a VDPV2 excretor was another healthy 3-year-old girl who received 2 doses of poliovirus vaccine; for the second dose received on 10 December 2020, IPV was used (Table 1). The child did not receive nOPV2. A fecal sample collected on 15 October 2021 contained VDPV2, and the result was obtained on 23 November 2021 due to the late delivery of material to NL. The next fecal sample collected only on 27 December 2021 was negative. Thus, virus excretion apparently ceased before December, but it remained undetected for 40 days until receiving the laboratory result.

Based on the genome sequence encoding the VP1 protein, these viruses were related to cVDPV2, which caused a poliomyelitis outbreak in Tajikistan. However, the isolates from the first child had 4.32% (sample collected 3 September 2021) and 3.99% (sample collected 13 September 2021) differences from the Sabin2 strain and the isolate from the second child (sample collected 15 October 2021) had 3.21%, which exceeds the 2.21–2.88% differences found in viruses isolated from patients in Tajikistan.

Children were identified in two different regions of Russia. For each case of VDPV2 isolation, a complex of anti-epidemic and preventive measures was organized in accordance with the sanitary legislation of Russia [18] and taking into account the WHO standard operating procedure for responding to the detection of poliovirus type 2 [29]. Children were isolated until a negative fecal test result was obtained. VDPV2 was not isolated from their close contacts.

### 3.2. nOPV2 Isolation

**Figure 1:**
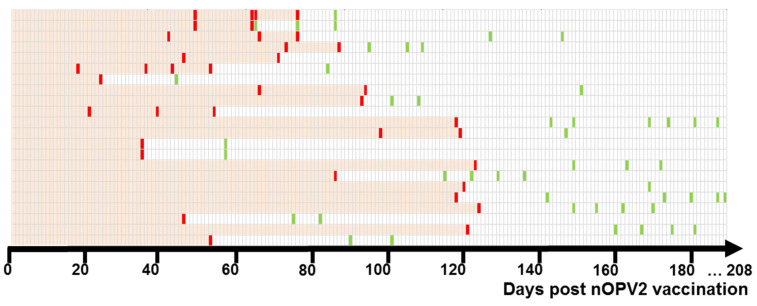
Days of nOPV2 derivatives isolation in stool samples of children with known dates of nOPV2 vaccination. Each horizontal line signifies one excretor. Day 0 was a known nOPV2 vaccination date. Excretion was estimated from day 0 to the last day with positive sample collection. Days with positive stool samples (virus isolations) are colored red, days with negative stool samples are green, and days of estimated excretion are pink.

The date of nOPV2 vaccination was known for 22 children who excreted nOPV2 viruses. The mean age of these children was 2.59 ± 1.47 years. The duration of the virus excretion was estimated from the day of vaccination to the day of the last positive sample collection, as the children could stop shedding between specimen collections. The shortest duration of virus shedding was 24 days (excretion ceased between 24 and 44 days), and the longest was 124 days (cessation of excretion between 124 and 149 days) (Figure 1).

The mean duration of shedding was 81.4 ± 32.9 (median 81) days. For 17 children known to have been vaccinated against polio before receiving the nOPV2 vaccine, the number of doses of poliovirus vaccine was 5.17 ± 1.7 doses (information about the type of vaccine—IPV or OPV—was not available). Five children were not vaccinated or had no information about previous vaccination. The duration of nOPV2 virus shedding by previously vaccinated (89.0 ± 27.9 days) and unvaccinated children (55.4 ± 38.5 days) did not differ significantly (*p* < 0.05). For 84 children, the date of receipt of nOPV2 was unknown. In this group, it was possible to determine the duration of nOPV2 shedding from the moment of border crossing into Russia. The duration of nOPV2 excretion for all these children was 57.8 ± 35.4 days. Nine children from this group, according to parents, received nOPV2 in June/August 2021. Allegedly vaccinated children excreted nOPV2 for 45.0 ± 32.9 days, and the remaining 75 allegedly unvaccinated children excreted for 59.3 ± 35.7 days. There was no significant difference in the duration of shedding between these two groups (*p* < 0.05).

**Figure 2:**
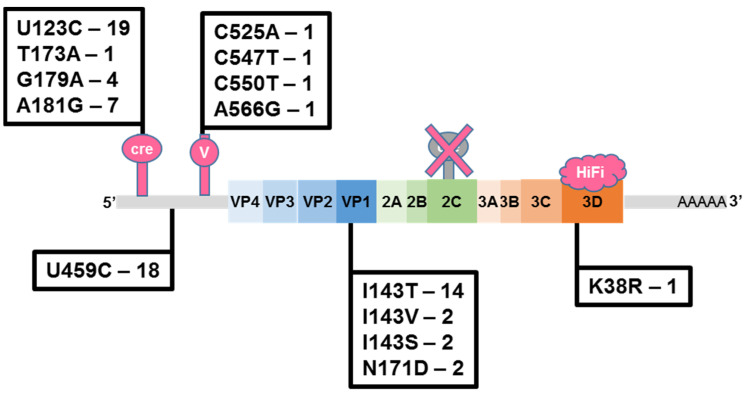
Substitutions in altered elements of the nOPV2 genome found in 24 investigated isolates detected in Russia. The number indicates the quantity of isolates with a given mutation. Genetically modified elements are indicated in pink (translocation of cre-element from 2C coding sequence to 5′UTR; modifications in domain V structure of 5′UTR; alternation of 3D coding sequence to increase RdRp fidelity).

Whole-genome sequencing was performed for 24 randomly chosen isolates. Sequence analysis and comparison with the nOPV2 vaccine strain revealed the following features: overall isolates contained 27.0 ± 14.9 (median 24) nucleotide mutations per genome and 4.46 ± 2.96 (median 3.5) per VP1 region. Genomes contained only point mutations and no signs of recombination with other enteroviruses (Figure 2). Most mutations were recorded in the cre-element transferred to the 5′UTR U123C—19/24—and further in domain IV 5′UTR secondary structure U459C—18/24. The latter structure was not altered and remained as in the Sabin2 sequence. Among the substitutions, in the structural part of the genome, the most common substitution was reversion in the VP1 protein I143T—14/24. A reversion in the polymerase gene, which reduced its fidelity, was detected only in one isolate—K38R.

The whole-genome analysis of the isolates allowed for their classification into two categories: 7 isolates scored 7 (no recombination, 6 or over nucleotide substitutions in VP1 region), and 17 isolates scored 8 (absence of recombination, up to 6 nucleotide substitutions in VP1 region). For isolates that scored 7, the number of mutations was 42.7 ± 16.0 (median 40) per genome and 8.29 ± 1.38 (median 8) mutations per VP1 region. For isolates that scored 8, the number of mutations was 20.6 ± 8.5 (median 23) per genome and 2.88 ± 1.69 (median 3) mutations per VP1 region.

**Figure 3:**
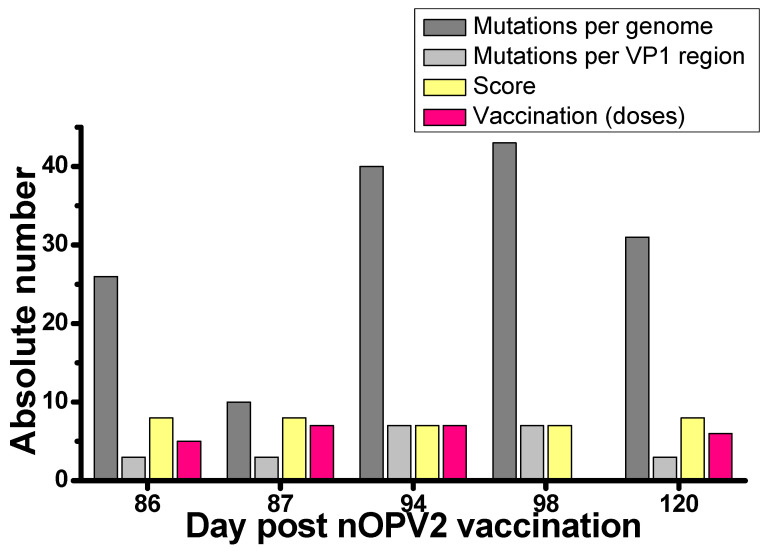
Number of nucleotide mutations, isolate classification, vaccination status and duration of excretion for five nOPV2 isolates with known dates of vaccination.

Five nOPV2 derivates were isolated from children with a known date of nOPV2 vaccination. The duration of the excretion of isolates that scored 7 were 94 and 98 days; the duration for isolates that scored 8 were 86, 87, and 120 days (Figure 3). Classification represents the number of mutations and corresponds to the duration of excretion. However, the isolate excreted for the longest period (120 days) contained fewer mutations than isolates excreted for 94 and 98 days. Four out of five children were fully vaccinated prior for poliomyelitis (5–7 doses). The unvaccinated child excreted nOPV2 virus for 98 days, and the isolate contained 43 nucleotide mutations per genomes. These numbers correspond with the ones for a vaccinated child excreting for 94 days. Overall, we did not observe any correlation between vaccination history and the time of excretion, although the number of analyzed cases is very low.

## 4. Discussion

The main efforts of the WHO Global Polio Eradication Initiative are now focused on combating the remaining foci of wild PV type 1 and cVDPV type 2 circulation [30], and there is slow but steady progress in this area [31]. However, the persistence of foci of circulation maintains the risk of the cross-border spread of these viruses, including to “polio-free” countries with high immunization coverage. The reality of this movement was illustrated in 2021–2022, when wild PV1, circulating in Pakistan in 2019, caused polio cases in Malawi and Mozambique on the African continent [32]. The potential for intercontinental spread was further confirmed by the isolation of genetically related type 2 cVDPVs in 2022 in Israel, the UK, the USA and Canada [32]. Since the certification of the European Region in 2002 [33], Russia has consistently maintained a high level of immunization coverage: 96–98% of children under 1 year of age received 3 doses of poliovirus vaccine [34]. However, the country has also experienced the importation of epidemically significant poliovirus variants [14,15,16,17]. Since 2018, the sequential vaccination schedule against polio in Russia has included 2 doses of IPV and 4 doses of bOPV, and since 2022, it has included 4 doses of IPV and 2 doses of bOPV. Therefore, overall population immunity to PV2 after the “switch” in April 2016 was ensured mainly due to IPV decreases [35], and, therefore, the potential for PV2 circulation increases, as has been demonstrated in countries that predominantly use IPV [32]. In this context, the screening of migrants from a country with an ongoing polio outbreak and using live poliovaccine type 2 is an important element of the epidemic welfare maintenance of the country and public health risk mitigation.

The isolation of cVDPV2 from migrant children without clinical manifestations of poliomyelitis was, in a certain sense, an expected event. The last VDPV2 was isolated in Tajikistan on 25 July 2021 from a polio case [36]. Both children shedding cVDPV2 entered Russia before the official end of the outbreak in April 2022. The date of entry of the first child practically coincided with the time of the last polio case in Tajikistan, but the second child entered Russia two months after the detection of the last cVDPV2 case (Table 1). This indicates that there was still an undetected circulation of cVDPV2 in Tajikistan, at least until mid-October 2021. However, it cannot be completely ruled out that the child could have been infected after entering Russia as a result of contacts in the migrant community. Moreover, in both cases, cVDPV2 was isolated 40–45 days after children’s arrival to Russia. During this time, the excretion of epidemically significant poliovirus remained undetected. Considering the children’s young ages, their circles of contacts were limited to the family members, but it was difficult to exclude the possibility of the virus “spreading” beyond the migrant community. Therefore, anti-epidemic measures (the isolation of virus-shedding individuals, the immunization of unvaccinated individuals from their immediate environment, the repeated laboratory testing of virus-shedding individuals, the examination of contacts, the mop-up immunization of children under 5 years of age) aimed at preventing the spread of the virus were justified and implemented.

The possibility of reversing and restoring the neurovirulence of vaccine strains is an integral property of live Sabin OPV [1,3]. The nOPV2 from genetically modified Sabin strains is designed to overcome this deficiency, which is now a “stumbling block” for the GPEI. Therefore, before the nOPV2 widespread introduction, numerous large-scale studies were conducted to investigate the immunogenicity, efficacy, and safety of this vaccine for different groups of recipients: children and adults [37,38,39,40,41]. The active nOPV2 use after WHO approval to combat cVDPV2 outbreaks confirmed its high effectiveness and safety [8]. A study of nOPV2 isolates collected in different countries after its use showed the high genetic stability of the vaccine virus and allowed for the conclusion that the risk of formation of new circulating viruses is very low [28]. However, the emergence of nOPV2-derived polioviruses responsible for paralytic cases, the formation of new genetic lines of cVDPVs derived from nOPV2, and their penetration beyond the vaccine application areas [8,42,43,44] has been described and indicates the need to continue to closely monitor the results of the new live polio vaccine, especially in terms of its genetic stability and safety.

Our observation strongly suggests that nOPV2-derived virus can easily be introduced into a country where this vaccine has not been used. Notably, one nOPV2 derivative was isolated from a child who came from Kyrgyzstan, which directly borders Tajikistan, in contrast to Russia, which does not have direct borders. The “presence” of the virus in Russia continued at least until 10 January 2022 (the date of the last isolation of nOPV2 from a child), i.e., at least 4 months after supplementary immunization activities using nOPV2 in Tajikistan from 30 August to 4 September 2021 [11]. Our study included a specific target group of migrant children who do not typically attend childcare facilities, so the possible circulation of nOPV2 may be considered limited to this group. Indeed, during AFP surveillance from AFP cases and their healthy contacts (in 2021 and 2022, 347 and 446 AFP cases were studied, through 309 and 987 contact persons, respectively), not a single nOPV2-derived virus was isolated. At the same time, during the environmental surveillance of poliovirus in Russia (17,350 wastewater samples were examined in 2021, 17,279 in 2022), two nOPV2-positive samples were detected in two different regions of the country, and in one of the regions, the virus from children had not been isolated. Obviously, the spread of the virus in the country was much wider than we had established (the virus was isolated from children in 37 regions). Notably, no nOPV2-derived virus was detected in a specific wastewater study undertaken as part of the nOPV2 clinical trials in Panama [45].

In studies examining the immunogenicity, efficacy, and safety of nOPV2, the period for fecal sampling was limited by the conditions of preclinical and clinical trials; it did not exceed 28 days [37,38,39,40,41]. We found that the duration of excretion of nOPV2-derived virus can last up to 4 months (on average 81.36 ± 32.9 days), does not depend on the previous history of polio vaccination and practically does not exceed the duration of shedding of Sabin type 2 [1]. As in the study of the genetic characterization of nOPV2 isolates obtained after immunization activities with nOPV2 in different countries [28], our results confirm the high genetic stability of nOPV2. It is noteworthy that in our study, the interval between vaccine receipt and fecal sample collection was slightly longer (120 days, Figure 3) than in the study by Martin et al. (81 days) [28].

A certain limitation of our study was insufficient or not entirely reliable information about the vaccination status of migrant children. We believe that this limitation did not have a significant impact on the conclusions from the study, but we believe that the issue of the need to provide documented information about vaccination should be taken into account by the relevant administrative structures of countries that receive migrants from epidemic-prone countries.

## 5. Conclusions

More than 30 years of GPEI experience have convincingly demonstrated the importance of additional types of surveillance for poliomyelitis [30]. In recent decades, many countries in the WHO European Region have faced significant numbers of migrants and refugees [46], including from countries where wild PVs and VDPVs were detected. Often, migrant children lack documentation of their vaccination history. Many host countries adhere to the practice of single vaccination followed by serological monitoring [47,48,49,50]. The investigation of stool samples is less common [51,52,53]. This requires significant organizational efforts and interaction with representatives of various local official services (migration, sanitary and epidemiological), especially when migrants are not in special places of stay but disperse throughout the country, as in Russia. However, such studies make it possible to identify the suspected introduction of poliovirus, conduct a risk assessment and take anti-epidemic measures if necessary. Monitoring the recovery of nOPV2 isolates has provided additional information about the genetic stability of the strains and has shown for the first time that the excretion of nOPV2-derived viruses can be prolonged. Such data are important for assessing the prospects for the use of live poliovaccines from genetically modified strain types 1 and 3 [54] and developing rational immunization strategies for poliovirus type 2. In this context, it should be noted that in addition to the “gold standard” of laboratory detection of poliovirus used in the WHO laboratory network [21], there is an increasingly clear need to develop and implement new standardized and inexpensive screening methods for the early detection of polioviruses, including viruses derived from novel poliovirus vaccines [30,55,56,57].

## Figures and Tables

**Table 1:** Information on VDPV2 isolation from excretors.

Patient	Number of Polio Vaccine Doses/Date of Last Vaccination	Entry to Russia	Date of Sample Collection (year 2021) and Investigation Result								
1	1/bOPV 9 Aug 2018	29 Jul 2021	3 Sep VDPV2	13 Sep VDPV2	1 Oct VDPV2	8 Oct VDPV2	15 Oct negative	22 Oct VDPV2	29 Oct negative	12 Nov negative	17 Nov negative
2	2/IPV 10 Dec 2020	20 Sep 2021	15 Oct VDPV2	27 Dec negative							

## Data Availability

The data are available in this article; raw data are available upon request.
